# Circulating Brain-Reactive Autoantibody Profiles in Military Breachers Exposed to Repetitive Occupational Blast

**DOI:** 10.3390/ijms252413683

**Published:** 2024-12-21

**Authors:** Shawn G. Rhind, Maria Y. Shiu, Oshin Vartanian, Catherine Tenn, Ann Nakashima, Rakesh Jetly, Zhihui Yang, Kevin K. Wang

**Affiliations:** 1 Defence Research and Development Canada, Toronto Research Centre, Toronto, ON M3K 2C9, Canada; maria.shiu@drdc-rddc.gc.ca (M.Y.S.); oshin.vartanian@drdc-rddc.gc.ca (O.V.);; 2 Faculty of Kinesiology and Physical Education, University of Toronto, Toronto, ON M5S 2W6, Canada; 3 Department of Psychology, University of Toronto, Toronto, ON M5S 2E5, Canada; 4 Defence Research and Development Canada, Suffield Research Centre, Medicine Hat, AB T1A 8K6, Canada; catherine.tenn@drdc-rddc.gc.ca; 5 The Institute of Mental Health Research, University of Ottawa, Royal Ottawa Hospital, Ottawa, ON K1Z 7K4, Canada; rakesh@drjetly.com; 6 McKnight Brain Institute, University of Florida, Gainesville, FL 32610, USA; zhihuiyang05@gmail.com (Z.Y.); kwang@msm.edu (K.K.W.); 7 Department of Neurobiology, Center for Neurotrauma, Multiomics & Biomarkers, The Neuroscience Institute, Morehouse School of Medicine, Atlanta, GA 30310, USA; 8 Center for Visual and Neurocognitive Rehabilitation, Atlanta VA Health Care System, Decatur, GA 30033, USA

**Keywords:** autoimmune antibodies, blast, blood–brain barrier, brain injury, breacher, anti-GFAP, anti-MBP, anti-pituitary, inflammation, repetitive subconcussive head impacts

## Abstract

Military breachers are routinely exposed to repetitive low-level blast overpressure, placing them at elevated risk for long-term neurological sequelae. Mounting evidence suggests that circulating brain-reactive autoantibodies, generated following CNS injury, may serve as both biomarkers of cumulative damage and drivers of secondary neuroinflammation. In this study, we compared circulating autoantibody profiles in military breachers (*n* = 18) with extensive blast exposure against unexposed military controls (*n* = 19). Using high-sensitivity immunoassays, we quantified IgG and IgM autoantibodies targeting glial fibrillary acidic protein (GFAP), myelin basic protein (MBP), and pituitary (PIT) antigens. Breachers exhibited significantly elevated levels of anti-GFAP IgG (*p* < 0.001) and anti-PIT IgG (*p* < 0.001) compared to controls, while anti-MBP autoantibody levels remained unchanged. No significant differences were observed for any IgM autoantibody measurements. These patterns suggest that repetitive blast exposure induces a chronic, adaptive immune response rather than a short-lived acute phase. The elevated IgG autoantibodies highlight the vulnerability of astrocytes, myelin, and the hypothalamic–pituitary axis to ongoing immune-mediated injury following repeated blast insults, likely reflecting sustained blood–brain barrier disruption and neuroinflammatory processes. Our findings underscore the potential of CNS-targeted IgG autoantibodies as biomarkers of cumulative brain injury and immune dysregulation in blast-exposed populations. Further research is warranted to validate these markers in larger, more diverse cohorts, and to explore their utility in guiding interventions aimed at mitigating neuroinflammation, neuroendocrine dysfunction, and long-term neurodegenerative risks in military personnel and similarly exposed groups.

## 1. Introduction

Military breachers, who are routinely exposed to low-level blast overpressure during training and operations, face occupational hazards with profound implications for brain health [1,2,3,4,5]. While the acute neurological effects of high-level blast exposure, such as concussion and traumatic brain injury (TBI), are well-documented, growing evidence highlights the cumulative and insidious effects of repeated low-level blast exposure on the central nervous system (CNS) [6,7,8,9,10,11]. Chronic exposure to blast waves is associated with transient neurological symptoms as well as enduring structural and functional brain changes, contributing to persistent neuroinflammation, long-term cognitive impairments, and an elevated risk of neurodegenerative conditions [12,13,14,15,16,17,18].

An emerging focus in neurotrauma research is the role of autoantibodies targeting brain-specific proteins—such as glial fibrillary acidic protein (GFAP), myelin basic protein (MBP), and pituitary (PIT) antigens—in the pathophysiology of repetitive head impacts and blast-induced neurotrauma [19,20,21,22,23,24,25,26,27]. These autoantibodies, generated in response to CNS injury, reflect adaptive immune-mediated processes triggered by repeated breaches of the blood–brain barrier (BBB) [28,29,30,31,32]. The BBB, which normally shields the CNS from systemic circulation, is particularly vulnerable to the mechanical forces and microvascular stress caused by blast waves [33,34,35,36]. When the BBB is compromised, CNS-specific proteins such as GFAP and MBP are released into the bloodstream, where they act as autoantigens. This exposure activates B lymphocytes, initiating an autoimmune response that results in the production of immunoglobulins (Ig) with an affinity for these normally sequestered self-antigens [37,38,39,40]. These autoantibodies such as anti-GFAP, anti-MBP, and anti-PIT may exacerbate CNS injury by amplifying neuroinflammation and perpetuating BBB dysfunction, creating a cycle of neural damage and immune activation [28,40,41,42].

Beyond serving as biomarkers of CNS injury, autoantibodies may actively contribute to secondary damage through neuroinflammatory and immune-mediated mechanisms [43,44,45]. GFAP, an astrocytic cytoskeletal protein released during glial injury and gliosis [46,47,48], has been implicated in astrocytic dysfunction and chronic neuroinflammation, hallmarks of blast-induced neurotrauma [22,26,49,50]. Similarly, MBP, which is critical for myelin integrity, is released during axonal damage and demyelination [51,52]. Anti-MBP autoantibodies have been shown to exacerbate myelin degradation, impair axonal function, and contribute to cognitive and motor impairments in blast-exposed individuals [37,53,54,55]. Additionally, autoantibodies targeting PIT antigens have been linked to neuroendocrine dysfunction, including posttraumatic hypopituitarism, a condition increasingly recognized in individuals with repetitive head trauma or blast exposure [56,57,58,59,60].

Autoantibodies offer valuable insights into the pathophysiological mechanisms underlying blast-induced neurotrauma and hold promise as minimally invasive biomarkers for diagnosing and monitoring CNS injury [31,61,62]. Elevated levels of IgG autoantibodies, particularly those targeting GFAP, MBP, and PIT antigens, are indicative of sustained antigenic stimulation and chronic immune activation, consistent with prolonged injury [55,63,64]. In contrast, IgM autoantibodies are associated more with acute-phase immune responses, with lower levels observed in chronic conditions like repeated blast exposure [24,65]. Understanding the distinct roles of IgG and IgM autoantibodies may provide critical insights into the progression of CNS injury and aid in identifying therapeutic targets [39,66].

The generation of autoantibodies against brain-specific proteins highlights the complex interplay between the immune system and CNS injury. While growing evidence demonstrates post-injury autoimmunity through the detection of various brain-specific autoantibodies in both preclinical and clinical studies, this area remains relatively underexplored in military personnel exposed to blast waves. This study examines circulating levels of IgG and IgM autoantibodies against GFAP, MBP, and PIT antigens in military breachers exposed to repeated low-level blast waves, compared to unexposed military controls. We hypothesized that chronic blast exposure would be associated with elevated autoantibody levels, indicative of BBB disruption, neuroinflammation, and immune-mediated CNS injury. By characterizing these autoantibodies, we aim to deepen the understanding of blast-induced neurotrauma, identify potential diagnostic and prognostic biomarkers, and inform therapeutic strategies to mitigate long-term neurological consequences in this high-risk population.

## 2. Results

### 2.1. Participant Demographics and Military Characteristics

The demographics, service histories, and occupational exposure characteristics of the blast-exposed Canadian Forces School of Military Engineering (CFSME) breacher instructors and range staff (*n* = 18; “Breachers”) and the unexposed Canadian Armed Forces (CAF) “Controls” (*n* = 19) are summarized in Table 1. The two groups were well-matched for age (mean ± SD: 32± years, range: 22–52), sex (89.5% male), lifestyle, and injury history. They also reported significantly greater career exposure to explosive blasts, although self-reported concussion histories were comparable between groups.

### 2.2. Autoantibody Levels in Military Breachers and Controls

#### 2.2.1. Anti-GFAP IgG and IgM

**Figure 1:**
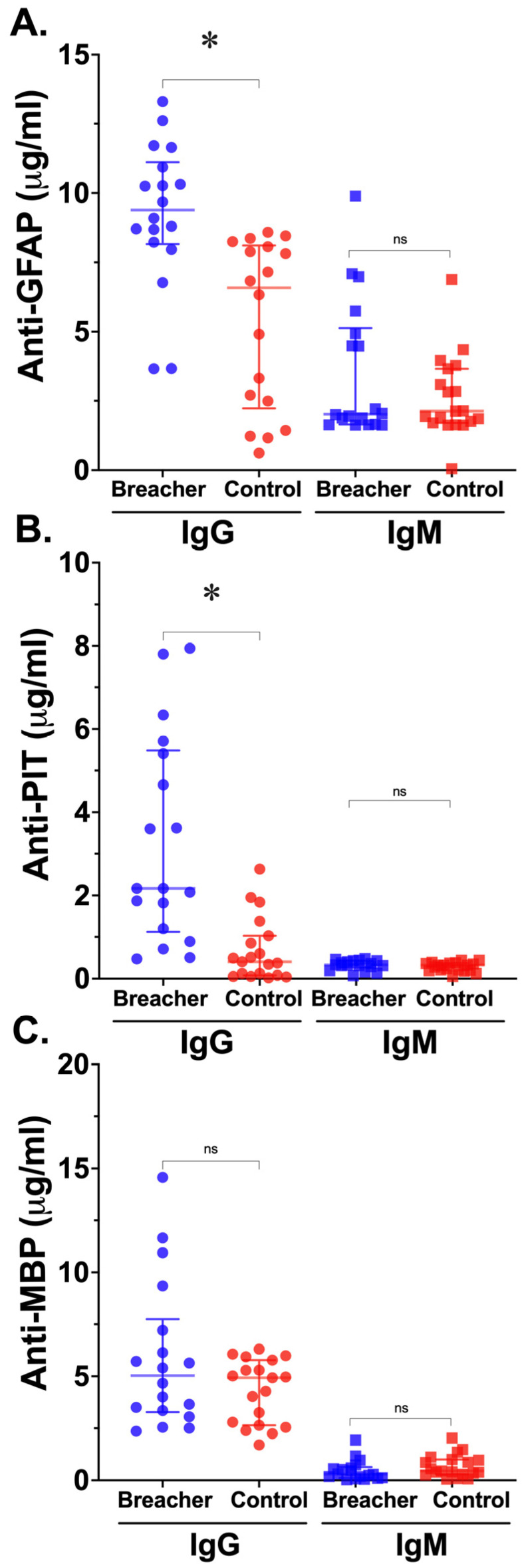
Circulating Autoantibodies in Breachers vs. Controls. Data are displayed as individual scatter plots showing the median and interquartile range (IQR; bars) for plasma concentrations (μg/mL) for each biomarker among blast-exposed Breachers (closed blue symbols) and unexposed Controls (closed red symbols) as indicated for IgG and IgM antibody isotypes; anti-glial fibrillary acidic protein (GFAP; (**A**)), anti-pituitary (PIT; (**B**)), anti-myelin basic protein (MBP; (**C**)). Mann–Whitney *U*-test significance vs. Controls: ns (not significant), * *p* < 0.001.

Military Breachers exhibited significantly elevated levels of anti-GFAP IgG compared to unexposed Controls (median [IQR]: 9.39 [7.9–10.5] μg/mL in Breachers vs. 5.31 [4.2–6.7] μg/mL in Controls, *p* < 0.0001, Figure 1A). In contrast, anti-GFAP IgM levels were not significantly (ns) different between Breachers and Controls (median [IQR]: 1.89 [0.9–4.9] μg/mL in Breachers vs. 2.13 [0.8–3.6] μg/mL in Controls, *p* = ns). These findings suggest a chronic or sustained immune response targeting astrocytic proteins, consistent with repeated BBB disruption.

#### 2.2.2. Anti-Pituitary IgG and IgM

Breachers also showed significantly elevated levels of anti-pituitary (PIT) IgG compared to Controls (median [IQR]: 2.17 [1.2–5.4] μg/mL in Breachers vs. 0.40 [0.19–3.6] μg/mL in Controls, *p* < 0.0001, Figure 1B). In contrast, anti-PIT IgM levels did not differ significantly between the groups (median [IQR]: 0.32 [0.7–1.1] μg/mL in Breachers vs. 0.31 [0.6–1.0] μg/mL in Controls, *p* = ns). These results indicate an adaptive immune response primarily mediated by IgG antibodies against pituitary antigens in Breachers, potentially implicating chronic neuroendocrine dysregulation.

#### 2.2.3. Anti-MBP IgG and IgM

Although anti-MBP IgG levels tended overall to be higher in Breachers compared to Controls, this difference did not reach statistical significance (median [IQR]: 5.03 [4.1–7.6] μg/mL in Breachers vs. 4.93 [3.5–5.0] μg/mL in Controls, *p* = ns, Figure 1C). Similarly, anti-MBP IgM levels were not significantly different between the two groups (median [IQR]: 0.30 [0.03–0.60] μg/mL in Breachers vs. 0.45 [0.6–1.0] μg/mL in Controls, *p* = ns). These findings suggest that while myelin-associated injury may occur in breachers, the immune response against MBP is less robust compared to GFAP and pituitary antigens.

## 3. Discussion

The present study provides evidence that circulating IgG autoantibodies against glial fibrillary acidic protein (GFAP) and pituitary (PIT) antigens are elevated in military breachers subjected to repetitive low-level blast overpressure. These findings add to a growing body of literature implicating chronic BBB disruption, sustained neuroinflammation, and adaptive immune activation as key features of repetitive blast-induced neurotrauma. By focusing on these two distinct protein targets—astrocyte-specific GFAP and pituitary-derived antigens—we extend our understanding of how repetitive blast exposure may perturb both glial integrity and the HPA axis, potentially leading to long-term neurological and neuroendocrine sequelae.

Our results are consistent with prior human and animal studies demonstrating that repetitive CNS trauma and blast exposure can trigger astrocytic dysfunction, reactive gliosis, and the release of astrocytic proteins into circulation [25,49,53,55,67]. GFAP serves as a sentinel intermediate filament protein of mature astrocytes, and its release into the bloodstream following BBB disruption confers antigenic properties that may initiate or sustain an adaptive immune response [27,68]. Elevated anti-GFAP IgG autoantibodies have been associated with autoimmune astrocytopathy, chronic neuroinflammation, and the cumulative effects of repetitive mild traumatic brain injury [42,69,70]. As such, the detection of circulating anti-GFAP IgG may reflect ongoing astrocytic damage and gliosis, positioning it as a candidate biomarker of cumulative astrocytic injury in repetitive blast exposure scenarios [71,72].

Similarly, the significant elevation of anti-PIT IgG in our Breacher cohort highlights the vulnerability of the HPA axis to blast-related injury [73,74]. Neuroendocrine dysfunction, including posttraumatic hypopituitarism, is increasingly recognized as a long-term consequence of repeated head trauma in both military and athletic populations [57,59,60,75]. Chronic immune responses against pituitary antigens could be driven by blast-induced BBB compromise, exposing otherwise sequestered pituitary proteins to the peripheral immune system and provoking a sustained autoimmune response [56,58,76]. This mechanism may help explain the heightened incidence of neuroendocrine disorders in individuals chronically exposed to low-level blasts [77,78,79,80,81]. Understanding the relationship between pituitary-targeted autoimmunity, blast dose, and clinical outcomes may inform both diagnostic strategies and therapeutic interventions aimed at mitigating long-term neuroendocrine sequelae [59,78,82].

Further supporting our findings in breachers, Harper et al. demonstrated that rodent models of blast-induced TBI develop serum autoantibodies against key CNS antigens, including GFAP and myelin, providing a mechanistic framework for the observed immune responses [26]. Complementary porcine studies have further shown that citrullinated GFAP and its breakdown products serve as neoantigenic epitopes, triggering sustained autoantibody production and driving a cycle of neuroinflammation and BBB disruption in chronic blast-induced neurotrauma [22,24,83,84,85]. Notably, these patterns also extend to human populations: military cohorts exposed to repetitive subconcussive blasts exhibit elevated anti-GFAP autoantibodies against neuroglia-specific and vascular proteins months after exposure, suggesting persistent astrocytic injury, ongoing gliosis, and long-term immune dysregulation [86].

Intriguingly, while we identified robust elevations in anti-GFAP and anti-PIT autoantibodies, we observed no significant increase in anti-MBP IgG or IgM in Breachers versus Controls. Given that MBP autoantibodies are implicated in demyelinating disorders such as multiple sclerosis [87,88] and often arise following more severe CNS insults [51,89,90], this absence suggests that repetitive low-level blast exposures may not produce the same extent of myelin injury as higher-intensity blasts [53,91,92,93]. Thus, the dose, frequency, and intensity of exposure likely shape the spectrum of CNS epitopes that become immunogenic, and defining these thresholds will be critical for understanding variability in autoantibody profiles across different blast exposure paradigms in operational and training environments [38,52,94].

Autoantibodies, particularly of the IgG isotype, are increasingly recognized as indicators of cumulative CNS injury in both human and animal studies [39,55]. In our findings, the preferential elevation of IgG autoantibodies suggests sustained antigenic drive and ongoing adaptive immune activation, contrasting with the IgM responses generally linked to acute injury [31,65,95,96]. This pattern of chronic autoantibody production is consistent with the progressive nature of repetitive blast-induced neurotrauma, involving repeated cycles of BBB disruption, antigen release, and immune sensitization [31,96,97]. Such persistent immune activity may amplify secondary injury pathways via prolonged neuroinflammation, ultimately destabilizing BBB integrity and increasing the likelihood of long-term neurological and neuroendocrine complications [22,24,26,53,86,98,99].

Our interpretation of these findings must be considered in light of several limitations. Our relatively small and demographically homogeneous cohort limits the generalizability of the results and may obscure subtle variations in autoantibody responses that could emerge in more diverse populations, including those differing by age, sex, or occupational exposure levels [100,101]. To address this, future studies should recruit larger, more heterogeneous cohorts to determine whether specific subgroups are particularly susceptible to blast-induced autoimmunity and to enhance the translational potential of these biomarkers [102]. Furthermore, our cross-sectional design precludes definitive conclusions about causality. Without longitudinal data, it is challenging to ascertain whether elevated autoantibody levels reflect acute responses, ongoing immune activation, or cumulative injury over time [4]. Long-term follow-up with serial sampling and comprehensive clinical monitoring will be essential to clarify the temporal dynamics of autoantibody production, map the emergence of clinical symptoms, and delineate how BBB compromise, immune sensitization, and CNS injury progression interact [28,103].

Expanding autoantibody profiling to include other CNS-specific targets, such as tau isoforms, amyloid beta, neurofilament light, or additional neural and vascular proteins, may provide a more comprehensive picture of the pathophysiology underlying blast-related brain injury [49,55,104,105]. Importantly, the selection of antigenic targets should be informed by clinical relevance. Integrating biomarkers with objective assessments of neurocognitive function, neuroimaging findings, and endocrine parameters would enhance our ability to link immune responses to functional outcomes [106,107,108]. Such an integrative approach would not only foster a deeper understanding of the underlying mechanisms of blast-induced neurotrauma but also increase the translational impact of these findings [109,110]. Ultimately, linking biomarker profiles to clinically meaningful endpoints may facilitate early risk stratification, inform targeted interventions, and guide the development of therapeutic strategies to prevent or ameliorate long-term neurological and neuroendocrine dysfunction [111,112,113].

It is also important to note that, although we cannot fully exclude minor contributions from non-CNS-derived sources of circulating GFAP, MBP, and pituitary peptides, such influences appear minimal and are unlikely to substantially alter the observed autoantibody profiles. Although these proteins are primarily associated with the CNS, limited expression can occur in select extracranial tissues, including Schwann cells, chondrocytes, testicular Leydig cells, enteric glia, podocytes, and liver stellate cells [114,115,116]. Similarly, while MBP is present in the peripheral myelin of the PNS, and pituitary antigens remain confined to the HPA axis [51,78], they generally remain sequestered under normal physiological conditions and do not enter circulation in significant amounts without substantial peripheral injury or systemic pathology [117,118,119]. Consequently, the patterns observed here are far more consistent with CNS-derived antigen release following BBB disruption than with significant extracranial contributions.

In healthy individuals, GFAP and MBP are typically undetectable or present only at trace levels in circulation [120,121]. Under normal conditions, robust immune tolerance mechanisms—such as central deletion, anergy induction, and regulatory T cell suppression—prevent these self-proteins from eliciting an autoimmune response [122]. Consequently, low-level or intermittent release from extracranial sources rarely breaches immune tolerance or triggers autoimmunity [123]. Moreover, the quantities of GFAP and MBP released under physiological conditions are substantially lower than those that follow CNS or PNS injury, particularly when accompanied by BBB compromise [25,124]. Pathological disruptions, such as those induced by repetitive blast exposure, can induce the mechanical and biochemical breakdown of the BBB [34,35], allowing CNS-specific proteins to enter the bloodstream and stimulate autoantibody production [1,7,50,57,91,98,125,126,127]. Combined, these considerations strongly support the interpretation that the elevated autoantibody responses observed in our Breacher cohort reflect CNS-derived antigenic release secondary to BBB disruption, rather than peripheral antigenic contributions.

Collectively, these findings underscore the emerging perspective that autoantibodies play an integral role in the pathogenesis of chronic CNS injury and potentially drive neurodegenerative processes. Repetitive blast exposure may amplify these pathological trajectories through sustained, low-grade inflammation and ongoing antigenic stimulation. The systematic profiling of autoantibody responses in exposed populations could serve not only as a predictive tool for early risk identification, but also illuminate novel mechanistic pathways by which chronic blast exposure disrupts neuroimmune homeostasis. By pinpointing key antigenic targets, clarifying their temporal dynamics, and linking these biomarkers to clinical outcomes, future research can help refine strategies aimed at mitigating the long-term neurological and neuroendocrine sequelae of repetitive blast injuries.

## 4. Materials and Methods

This study was conducted in accordance with the ethical standards of the Helsinki Declaration and approved by the Defence Research and Development Canada (DRDC) Human Research Ethics Committee (Protocol No. 2016-006). Written informed consent was obtained from all participants following a detailed briefing on the research procedures and associated risks.

### 4.1. Study Population and Experimental Design

This prospective, cross-sectional cohort study included 18 CFSME breacher instructors and range staff (“Breachers”) with extensive occupational exposure to explosive blast overpressure. A comparison group of 19 unexposed, age- and sex-matched CAF members (“Controls”) with similar operational experience and occupational stressors but no history of blast exposure was also recruited. Direct quantification of blast overpressure was not feasible; however, Breachers participated in 8–20 breaching courses annually, with 1–2 range days per week, each involving exposure to over six blast events per day. Participants provided their demographic and medical history, completed occupational and exposure-related questionnaires, and underwent self-reported symptom assessments alongside standardized neurobehavioral assessments, as described previously [4,128]. Exclusion criteria included diagnosed acquired brain injury, neurological or psychiatric disorders, learning disabilities, and drug abuse. All testing was performed at the same time of day and participants had not been exposed to blasts for a minimum of 48 h and were asked to refrain from strenuous physical activity for at least 24 h prior to testing.

### 4.2. Blood Collection and Processing

Fasting venous blood samples were collected by trained and experienced medical technologists into 10-mL K2EDTA vacutainers (BD Vacutainer^®^, Franklin Lakes, NJ, USA). Samples were centrifuged at 1600× *g* for 15 min at 4 °C, and plasma was aliquoted and stored at −80 °C for later analysis. All samples were processed uniformly and at the same time of day to minimize variability.

### 4.3. Measurement of Autoantibodies by Immunoassay

The detection and quantification of autoantibodies against GFAP, MBP, and pituitary antigens in plasma were performed using a customized enzyme-linked immunosorbent assay (ELISA) method, adapted from established protocols [22,55,98]. Briefly, custom 96-well ELISA plates were coated with antigens from bovine hypothalamic lysate (cat#0613, Science Cell Research, San Diego, CA, USA) or bovine pituitary extract lysate (Sigma, cat#P1476) (2 µg/well) or 50 ng/well purified human full-length GFAP protein (#DXAG-001, Dx-Sys, Mountain View, CA, USA) or human MBP protein (#M0689-1MG, Millipore-Sigma, Burlington, MA, USA) in 100 µL of 50 mM sodium carbonate-bicarbonate buffer (pH 9.6) for a minimum of 4 h and up to overnight at 4 °C. After plate preparation, 1 µL of human serum sample was mixed with 99 µL of StartingBlock buffer and transferred to each well (1:100 dilution) with incubation at 4 °C overnight with shaking. Plates were washed again 4 times with Tris Buffered Saline with 0.05% Tween 20 (TBST) wash buffer. A total of 100 μL of HRP-conjugated anti-human IgM (catalog no. 109-036-129; Jackson Immunoresearch, West Grove, PA, USA) or IgG (catalog no. 109-036-098; Jackson Immunoresearch) antibodies, diluted 1:10,000 in blocking buffer, was added to the wells. These secondary antibodies are specific to either human IgM or human IgG, respectively. Plates were incubated at 25 °C, with shaking for 45 min. After plate washing 4 times with TBST, 100 µL tetramethylbenzidine (TMB) substrate was added to develop color for 15 min. Stop Solution (100 µL; 1 dilution H_2_SO_4_ (0.61 M, TMB stop solution (catalog no. N600; Thermo Fisher Scientific, Waltham, MA USA)) was then added.

Optical density (OD) was measured at 450 nm with a reference wavelength of 540 nm using a Tecan Infinite M200 Pro ELISA reader. Standard curves for human IgG and IgM were included on each plate for quantification, and results were expressed in μg/mL after applying appropriate dilution factors. Intra-assay and inter-assay coefficients of variation (CV) were 5–10% and 15–20%, respectively, ensuring reliability and reproducibility. This sensitive method enabled the robust quantification of anti-GFAP, anti-MBP, and anti-PIT autoantibodies, providing critical insights into immune responses in blast-exposed military personnel and supporting their potential utility as biomarkers for CNS injury.

### 4.4. Statistical Analysis

All data underwent outlier analysis and assessment for normality and variance assumptions. Continuous variables are presented as mean ± standard deviation (±SD) for normally distributed data or as median with interquartile range (IQR) for non-normally distributed data, while categorical variables are expressed as frequencies and percentages. Group differences for normally distributed continuous variables were assessed using independent two-sample *t*-tests; for non-normally distributed variables, the Mann–Whitney *U*-test was applied. Mean difference scores for each continuous variable were calculated with 95% confidence intervals, and Cohen’s *D* effect sizes were computed using resampled means and pooled standard deviations. For categorical variables, group differences were evaluated using the chi-square (Χ^2^) test based on the percentage of individuals classified into each outcome group. Biomarker values were included for statistical analysis only if replicate measurements exhibited a coefficient of variation (CV) below 20% and were within the assay’s specified detection limits. Statistical significance was defined as a false discovery rate (FDR)-corrected *p*-value of <0.05. All analyses were conducted using GraphPad Prism 10 software (version 10.2.3, GraphPad Software, LLC, San Diego, CA, USA).

## 5. Conclusions

In conclusion, this study highlights the potential of circulating anti-GFAP and anti-PIT IgG autoantibodies as biomarkers of chronic CNS injury in military breachers exposed to repetitive low-level blasts. These results deepen our understanding of the underlying pathophysiological processes—encompassing BBB disruption, astroglial and pituitary vulnerability, and sustained immune activation—that drive blast-induced neurotrauma. Moving forward, studies involving larger, demographically diverse cohorts, longitudinal data collection, comprehensive clinical assessments, and expanded biomarker profiling will be critical to validating and refining these findings. Such efforts will help establish autoantibody signatures as valuable tools for early diagnosis, clinical monitoring, and targeted intervention strategies aimed at mitigating the long-term consequences of repetitive blast exposure.

## Figures and Tables

**Table 1:** Demographics and Service History.

Variables	Controls (*n* = 18)	Breachers (*n* = 19)	Mean Difference (95% CI)	Cohen’s D	*p* *
Age	32.5 (7.2)	33.3 (7.7)	0.7 (−4–5.3)	0.18	0.722
Sex (*n*, % male)	17 (0.9)	17 (0.9)	--	--	--
Years of Service	6.6 (5.6)	13.2 (6.6)	6.5 (3.4–10.4)	1.6	**<0.001**
Years of Explosives	0.5 (1.3)	11.1 (6.3)	10.6 (8.2–13.3)	3.5	**<0.001**
Years Breaching	0 (0)	7.2 (4.4)	7.2 (5.4–9.3)	3.5	**<0.001**
Warzone Deployment	0 (0)	11 (64.7)	68.3 (47.4–89.5)	2.9	**<0.001**
Blast Exposure	2 (10.5)	19 (100)	89.6 (73.7–100)	6.5	**<0.001**
Concussion	5 (26.3)	8 (44.4)	21.9 (−5.3–47.4)	0.7	0.08

Continuous data are presented as mean ± standard deviation (±SD), while categorical data are summarized as frequencies and percentages. * Statistical significance for continuous variables was assessed using an independent two-sample *t*-test, with *p*-values corrected for a false discovery rate of 0.05. For categorical variables, group differences were evaluated using the chi-square (Χ^2^) test based on the percentage of individuals classified into each outcome.

## Data Availability

The datasets presented in this article are not publicly available because they contain information that could compromise the privacy of military research participants. Requests to access the datasets should be directed to the corresponding author.
